# Sexual Dysfunctions of HIV-Positive Men: Associated Factors, Pathophysiology Issues, and Clinical Management

**DOI:** 10.1155/2011/854792

**Published:** 2011-10-20

**Authors:** Marco de Tubino Scanavino

**Affiliations:** Sexuality Studies Program (ProSex), Department and Institute of Psychiatry, Hospital das Clínicas da Faculdade de Medicina da Universidade de São Paulo, Rua Dr. Ovidio Pires de Campos, 785, 05403-010 São Paulo, SP, Brazil

## Abstract

Sexual dysfunctions in HIV-positive men are associated with an increase in risky sexual behavior and decreased adherence to antiretroviral drug regimens. Because of these important public health issues, we reviewed the literature on the pathophysiology, associated factors and clinical management of sexual dysfunction in HIV-positive men. The goal was to investigate the current research on these issues. Literature searches were performed in June 2011 on PubMed, Web of Science, and PsycInfo databases with the keywords “AIDS” and “sexual dysfunction” and “HIV” and “sexual dysfunction”, resulting in 54 papers. Several researchers have investigated the factors associated with sexual dysfunction in HIV-positive men. The association between sexual dysfunction and antiretroviral drugs, particularly protease inhibitors, has been reported in many studies. The lack of standardized measures in many studies and the varying study designs are the main reasons that explain the controversial results. Despite some important findings, the pathophysiology of sexual dysfunction in the HAART era still not completely understood. Clinical trials of testosterone replacement therapy have shown the treatment to be beneficial to the improvement of sexual dysfunctions related to hypogonadism. However, there are not enough psychological intervention studies to make conclusions regarding the therapeutic effects of psychotherapy.

## 1. Introduction

Highly active antiretroviral therapy (HAART) has previously been shown to provide the best clinical management for HIV-infected patients, as it decreases the prevalence of hypogonadism and advanced HIV disease, which are the principal causes of sexual dysfunction in people infected with HIV [1, 2]. However, the prevalence of sexual dysfunctions in the years since the advent of highly active antiretroviral therapy (HAART), which varies according to the cultural and methodological issues of the studies in question, includes high rates of erectile dysfunction (ED) (9–74%), ejaculatory disturbances (36–42%), and low sexual desire (LSD) (24–73%) [2].

In an English clinical study conducted in 2001 with 78 HIV-positive gay men, 69% reported one or more sexual problems. ED was reported by 38% of the men, which rose to 51% in the context of trying to use condoms. Loss of interest in sex was reported by 41%, and 24% experienced delayed ejaculation [3]. From October 2002 to January 2003, a case-control Brazilian study nested in a cross-sectional population of individuals with AIDS found that almost 50% of the men reported ejaculatory symptoms, 33% reported ED, and 12% reported dyspareunia [4]. 

After receiving the diagnosis of HIV infection, it is common for people to experience negative moods and decrease the frequency of sexual activity, and those who remain sexually active most likely do so without adequate protection [5]. Adherence to safe sex practices after HIV diagnosis may have a negative impact on the sexual functioning of most subjects [6]. Individuals who are partnered are significantly more likely to maintain sexual activity than those who do not have a partner [7]. 

In a study with a convenience sample of 156 HIV-positive gay men and 155 HIV-negative gay men selected from the community and the internet who were matched for age and unprotected anal intercourse (UAI), the HIV-positive men were more likely to report ED and higher scores on the inhibition of arousal due to the threat of performance failure [8]. The presence of ED associated with condom use has been reported by gay men. Risk cognitions such as wanting to lose oneself in sex, placing the responsibility for condom use to the active partner, and perceptions that condoms interfere with pleasure were significantly more likely to be endorsed by those who reported ED associated with condom use [3].

In addition, it must be considered that individuals who acquire HIV through sexual or parenteral (excluding blood transfusions) routes of infection are already part of a population that is at higher risk for sexual dysfunction, as many risk factors for HIV are also linked to the occurrence of sexual dysfunction such as conflicts with one's sexual orientation or sexual identity, depression, and psychological problems related to self-image [9, 10].

Moreover, sexual dysfunction has an impact on the quality of life and very often leads to negative attitudes on the part of the individual, including poor adherence to antiretroviral regimens and to safer sex strategies [11–13]. HIV-infected people with sexual dysfunction who are nonadherent to antiretroviral regimens have an increased risk of transmission of drug-resistant strains because of higher-risk sexual behavior, higher HIV RNA concentrations in semen and cervical secretions [14], and inappropriate use of phosphodiesterase-5 (PDE-5) inhibitors without medical recommendations with higher likelihood of negative interaction with antiretrovirals [11, 12, 15].

Because of the increases in risky sexual behavior and decreased adherence to antiretroviral drugs reported in HIV-positive men with sexual dysfunctions, we decided to review the literature on the pathophysiology, associated factors, and clinical management of sexual dysfunctions in HIV-positive men. The goal of this review is to investigate the current research on these issues.

## 2. Methods

Literature searches were performed in June 2011 on the PubMed, Web of Science, and PsycInfo databases with the keywords “AIDS” and “sexual dysfunction,” which resulted in 36, 11, and 21 articles, respectively. These searches were also performed with the keywords “HIV” and “sexual dysfunction,” which resulted in 56, 12, and 22 articles, respectively. The abstracts were reviewed and the inclusion criteria required that the studies were clinical studies on the associated factors, pathophysiology, and clinical management of the sexual dysfunctions of HIV-positive men, and that they were published in English. Posters and abstracts of presentations in congress or scientific meetings were excluded from our search. Firstly, 20 studies were included and reviewed. After searching the reference lists of these papers, 34 more studies were reviewed, resulting in a total of 54 papers.

## 3. Results

Fifty-four papers on the associated factors, pathophysiology, and clinical management of sexual dysfunction in HIV-positive males were reviewed. 

### 3.1. Associated Factors

There are four important factors associated with sexual dysfunction in HIV/AIDS patients: mental, hormonal, pharmacological, and other morbid conditions.  Table 1 lists the studies on these associated factors. 

#### 3.1.1. Mental Factors

A representative French study on 1,812 HIV outpatients who were being treated for HIV and reported having had at least one sexual partner in the 12 months prior to the survey showed an association between sexual difficulties and knowing more than four people who lives with HIV/AIDS, perceiving side effects as very disturbing, reporting lipodystrophy-related symptoms and having a high HIV-discrimination score in the prior 12 months. Moreover, CD4 cell levels, viral load titers, and CDC stage, and current HAART treatment at the time of the study were not associated with sexual difficulties. The authors recommended psychological support for HIV patients in order to improve the sexual aspects of their lives [17]. Feelings of guilt from having acquired HIV via sexual transmission may be a mental factor that negatively influences the sexual response. This psychological factor may explain some reports of higher rates of sexual dysfunctions in homosexual and bisexual men [26, 27]. 

Depression is one of the most important mental factors associated with sexual dysfunctions [28]. A study on 300 HIV-infected men found that older age and depression were associated positively with ED, and higher CD4 cell counts were associated negatively, then, and higher CD4 was considered protective regarding ED [18].

#### 3.1.2. Hormonal Factors

Hypogonadism was one of the most frequent causes of sexual dysfunction before HAART. Currently, HIV-infected individuals may have higher testosterone levels than noninfected individuals. 

The prevalence of hypogonadism has been lowered with the introduction of HAART, but it still remains the most common endocrine disorder of HIV-infected men [29].

Moreover, estradiol is often higher in men (50% of them) on HAART, possibly due to augmented peripheral conversion of androgens to estrogens in lipid tissues [30, 31]. However, the role of estradiol in HIV-related sexual dysfunctions is not clear. The expected decreased levels of gonadotropin hormones in the blood were not observed [24], and one study observed improved sexual function despite higher blood levels of estradiol [23]. Hyperprolactinemia may be associated with sexual dysfunction as it decreases the release of gonadotropins and has been found in some HIV-positive individuals. However, one study found no difference in prolactin levels between patients with and without sexual difficulties [23].

#### 3.1.3. Pharmacological Factors

Table 1 lists the studies on the association between antiretrovirals, particularly protease inhibitors, and sexual dysfunction.

Ejaculatory dysfunction has been shown to be associated with the use of didanosine [9] as well. Moreover, HIV-infected individuals use many other medications that are associated with decreased sexual responses. Medications such as ketoconazole, fluconazole, ganciclovir, megestrol, methadone, and cimetidine may decrease testosterone levels and cause sexual dysfunction [6, 32]. Antihypertensives, diuretics, hypolipemics, benzodiazepines, antidepressants, and antipsychotics are also associated with sexual dysfunctions [19, 32–34].

#### 3.1.4. Comorbid Conditions

Some morbid conditions are common in HIV-infected individuals and some of them are often associated with sexual dysfunction, such as hepatopathy, diabetes, hyperlipidemia, hypertension, vascular disease, and alcohol dependence [35].

Peripheral neuropathy is a well-documented complication of both HIV infection by direct viral toxicity and HAART, most notably with thymidine analog nucleoside reverse transcriptase inhibitors. There is a significant association in HIV-positive men between peripheral neuropathy and delayed ejaculation [21].

Another study on 12 patients with ED reported neuropathy of the sacral region was associated with protease inhibitor use, particularly indinavir [26].

### 3.2. Pathophysiological Issues

One prospective, age- and sex-matched study in Nairobi on 25 patients (7 with asymptomatic HIV, 8 with AIDS-related complex, and 10 with AIDS) and 25 controls was evaluated for its autonomic functioning. Substantial autonomic dysfunctions were seen in AIDS patients relative to controls, and mild abnormalities in the majority of HIV-infected patients were found [36]. This finding could explain the association between delayed ejaculation and peripheral neuropathy [30]. The physiological process of ejaculation is under autonomic control via the hypogastric (sympathetic) and pudendal (parasympathetic) nerves [30].

Another relevant point regarding the pathophysiology of the sexual dysfunctions of this population is that many patients who are on HAART to treat their HIV infection and have lipodystrophy also have elevated estrogen levels and complain of low sexual desire. As a possible pathological mechanism, one can consider an increase in the number of fibroblasts and macrophages present in lipoatrophic areas that could convert testosterone to estrogen by intracellular aromatization. This process is known to be enhanced by increased levels of tumor necrosis factor, interleukin 6 (IL-6), and hydroxycorticosteroids present in many patients with HIV lipodystrophy [31]. 

In addition, a study on rabbits found estrogen receptors in the cavernous body of the penis and found pathophysiological changes in erectile function when rabbits were treated with continuous estrogen [37]. Another study of older men found that the balance between testosterone (decreased) and estradiol (increased) is associated with ED [38].

Anecdotal reports suggest an association between protease inhibitor use and sexual dysfunctions, but only a few studies have found specific evidence for the link. These studies found a possible effect of the protease inhibitors on the binding of the steroids hormones with their receptors [39] or causing the blockade of androgen receptor [40], both actions could be related to sexual difficulties associated with protease inhibitors.

### 3.3. Clinical Management

The clinical management of sexual dysfunctions in HIV/AIDS patients involves clinical assessment, pharmacotherapy, psychotherapy, and psychoeducational approaches to safer sex.  Table 2 lists the clinical management studies.

#### 3.3.1. Clinical Assessment

In order to investigate the sexual functioning of HIV-infected people, the first point to consider is ensuring an appropriate doctor-patient relationship [46]. It is important that professionals maintain an open-minded attitude and be free of judgments.

**Clinical History**

The clinical history contains the assessment of the patient's immunological conditions, comorbidities, and medications. Severe immunological damage may indicate an AIDS diagnosis. Poor health undermines the overall physical condition and the sexual response. Nevertheless, hypogonadism should be investigated. Upon physical examination, gynecomastia and testicular atrophy may indicate hypogonadism [47]. Hypogonadism is defined by low levels of testosterone (<300 nh/dL) in the early morning, with associated clinical manifestations, including sexual dysfunction, weight and muscle mass loss, fatigue, depressed mood, and anemia [29].

**Sexual History**

When sexuality is repressed, it can be difficult for individuals to fully experience sex and love in adolescence and young adult life [48]. These experiences are fundamental to the sexual maturation process. When it fails because of sexual repression and possible internal conflicts regarding living the sexuality experience, it can undermine the sexual response. Other factors in an individual sexual history may be relevant to their experience of sexual dysfunction like sexual violence suffered during childhood and adolescence, which can also lead to serious psychological effects and sexual consequences [48–50].

An individual's first sexual experiences with boys or girls, his/her first complete sexual relationship, and masturbation are all significant steps in the sexual maturation process, which involves gaining knowledge about one's body (erogenous zones) and of the bodies of others. When a person has sexual difficulties in their early sexual experiences that they are not prepared to deal with, these experiences can promote a negative attitude regarding sex; new experiences will be avoided, thus undermining sexual maturation [51].

A person with sexual inexperience is at higher risk for sexual dysfunction [51], and, in turn, a person with sexual dysfunction is at higher risk for unsafe sexual behavior [47], even if infected by HIV.

Some specific gender issues are also important and should be investigated. For men, homosexual orientation presents a special vulnerability for sexual dysfunction, perhaps because of the process of accepting sexual orientation, the difficulties in dealing with poor acceptance by family and society, and problems with gender identity [52]. Some studies have reported higher rates of sexual dysfunction in HIV-infected men who have sex with men [3].

**Characteristics of Organic or Psychogenic Sexual Dysfunctions**

It is also relevant to distinguish between characteristics of organic or psychogenic sexual dysfunction (Table 3) [23, 53–55]. Considering HIV infection, we could conclude that individuals who are seropositive but are in good health probably have sexual dysfunctions due to a psychogenic etiology, and individuals with poor immunological conditions or an AIDS diagnosis probably have sexual dysfunctions that are due to organic factors.

**Measures**

Some standardized instruments for the quick assessment of sexual responses can be used, as health practitioners often find it difficult to investigate the sex lives of their patients. For males, there is the International Index of Erectile Function (IIEF), which addresses the relevant domains of male sexual function (erectile function, orgasmic function, sexual desire, intercourse satisfaction, and overall satisfaction). This instrument is psychometrically tested and readily self-administered in research or clinical settings [56].

**Laboratory Assessment**

Laboratory assessment may involve a screening of sexual hormones including testosterone, estrogen, estradiol, prolactin, and gonadotropin. It is important to check the serum-free testosterone level or the levels of sex-hormone-binding globulin because they are usually increased in HIV-infected individuals [57]. Preliminary evaluations can be accomplished by determining the free testosterone level because the total level may be falsely reassuring due to an elevation in sex-hormone-binding globulin, the principal binding protein for testosterone [29].

When organic etiology is suspected, more comprehensive evaluations can take place, such as Doppler ultrasonography to assess possible arterial obstruction or nerve conduction studies to assess possible cases of neuropathy.

#### 3.3.2. Pharmacotherapy

**Antiretrovirals**

All antiretroviral drugs are associated with different degrees of sexual dysfunction. The highest rates of dysfunction are associated with indinavir and the lowest with nevirapine [58].

If medication is the principal factor leading to sexual dysfunction, one can try another drug that has less influence on sexual response, such as nevirapine [2, 58] or atazanavir [59].

**Phosphodiesterase-5 Inhibitors**

The use of PE-5 inhibitors is highly recommended in male sexual dysfunction [55], but one should be careful of drug interactions with antiretrovirals, particularly with protease inhibitors (especially ritonavir), the nonnucleoside reverse transcriptase inhibitor delavirdine, and ketoconazole. Because all of these drugs are metabolized by the cytochrome P-450 system, the dosages of PE-5 inhibitors should be reduced [47, 60]. In this situation, the recommended dosage of sildenafil is 25 mg every 48 hours (also reduced to 2.5 mg every 72 hours when also using ritonavir), of vardenafil is 2.5 mg every 24 hours, and of tadalafil is 10 mg every 72 hours [60–63].

Pharmacokinetic studies have demonstrated that sildenafil does not increase the blood levels of protease inhibitors, but indinavir, saquinavir, and ritonavir [60, 63] significantly increase sildenafil levels. There is, therefore, a risk of drug interaction between sildenafil and protease inhibitors, leading to hypotension and possible death in one case [64].

PE-5 inhibitors with longer half-lives may increase the risk of adverse effects for longer periods of time when combined with protease inhibitors. The use of sildenafil may be preferred because of its shorter half-life [29].

Poppers (amyl nitrate) are contraindicated by male users of PE-5 inhibitors because they lower blood pressure, especially in combination with PE-5 inhibitors [65].

**Testosterone Replacement**

If the patient meets the diagnostic criteria for hypogonadism, there are some options for testosterone replacement. Multiple formulations exist, ranging from intramuscular to transdermal and buccal administration. The latter avoids first-pass metabolism and results in more sustained serum testosterone concentrations [29].

Testosterone replacement is not prescribed for HIV patients without observed decreases in free testosterone blood levels because no improvements of sexual dysfunctions have been reported in this condition and the risks of treatment do not outweigh the benefits [2]. Testosterone replacement therapy can sometimes be problematic even in hypogonadal males. One study reported three HIV-infected patients with ED who presented low testosterone and sex-hormone-binding globulin (SHBG) despite receiving long-term oxandrolone in addition to testosterone replacement therapy and HAART.

Discontinuation of oxandrolone led to the normalization or improvement of testosterone levels in all three patients, with symptomatic improvement in one patient. The authors hypothesized that the first-pass metabolism of orally administered oxandrolone may decrease the hepatic synthesis of SHBG, allowing exogenously supplied testosterone to be excreted [41].

In addition, testosterone replacement shows good results in improving the sexual dysfunction of most of hypogonadal HIV-infected individuals [44, 66–69]. The use of a topical testosterone gel [43] was shown to improve sexual function and also caused heightened erythropoiesis, weight gain, prevention of bone loss, increased energy, and improved mood [29].

**Letrozole**

Finally, some improvement in sexual desire has been reported in a few patients on HAART who were treated with letrozole, an aromatase inhibitor that inhibits the conversion of testosterone to estradiol. Thirteen men who have sex with men who were on HAART with LSD and also had raised estradiol levels were randomly selected to receive either parenteral testosterone or letrozole for six weeks. Standardized instruments demonstrated improvements in desire and the frequency of sexual acts in both treatment arms [42].

#### 3.3.3. Psychotherapy

The psychotherapeutic approaches for the treatment of sexual dysfunction of HIV-infected people involve supportive and psychosexual therapy.

**Supportive**

If the most relevant factor leading to sexual dysfunction is psychogenic, one can use supportive therapy in the period immediately after HIV diagnosis. Therapy should focus on demystifying the stigmas associated with HIV/AIDS as a lethal disease that is associated with non-conventional sexual behavior. The supportive approach would diminish fear and guilt and help individuals deal with discrimination [17].

**Psychosexual**

Psychosexual therapy such as sensate focus or masturbatory training is indicated when the individual's HIV seropositivity has already been accepted psychologically but sexual dysfunctions still remain. The approach is the same as that used for nonHIV-infected individuals with sexual dysfunctions.

#### 3.3.4. Psychoeducational Approach to Safer Sex

A psychoeducational approach to safer sex is offered concomitant with the treatment of the sexual dysfunction. The approach always involves the patient and his or her partner. Safer sex counseling is fundamental for explaining the risks associated with contact with different strains of HIV, which favors the development of the resistance to antiretrovirals. 

Finally, a psychoeducational approach should encourage lifestyle modifications including safer sex, exercise, modifications of cardiovascular risk factors, and education about the effects of recreational drug use [47].

An essential part of managing sexual dysfunction in HIV patients is counseling about maintaining safer sexual practices to minimize the risk of acquiring other sexually transmitted infections and transmitting HIV to others. It must be emphasized that having an undetectable plasma HIV viral load does not mean that an individual is no longer able to pass on the virus.

There is good evidence that the HIV viral load in seminal fluid is independent of plasma HIV-1 RNA levels [70]. Even if intercourse occurs between HIV-positive concordant partners, the risks of sexual infections, transmission of drug-resistant HIV, and superinfection remain [70].

## 4. Discussion

As Table 1 shows, most (12/15) of the clinical studies on HIV-positive male sexual dysfunctions took place from 1998 to 2007. The majority (11/15) addressed the role of HAART or hormonal changes in the sexual dysfunctions of HIV-positive men.

A possible role of antiretroviral drugs in the generation of sexual dysfunction is controversial. Whereas some studies have supported a role of antiretroviral therapy in sexual dysfunction [19, 23, 71], other studies have not found such an association [3, 18, 72]. In the HAART era, there are high rates of sexual dysfunction despite general health improvements. 

Different methodological designs and the use or lack of standardized instruments are responsible for the controversial results regarding a possible association between sexual dysfunction and antiretrovirals. Just 4 of 15 studies (Table 1) used standardized measures in their evaluations of sexual dysfunctions. The different methodologies likely contribute to the varying results. 

In contrast to depression, which was addressed by most of the studies, other psychogenic factors were not often investigated (Table 1). 

In Table 2, we can see the scarcity of studies on the psychological interventions for sexual dysfunctions in HIV-positive individuals that aim to investigate the evidence for the clinical management of psychogenic sexual dysfunctions. Just one of the six studies addressed psychological treatment. Curiously, this study reported that the majority of the 34 HIV-positive homosexual men experienced an improvement of their sexual function. The problems were solved for 16 (76%) and improved for 3 (14%), whereas only 2 (10%) reported no change. However, the physical methods of treatment were more effective than psychological interventions [27] in this population.

Sexual functioning before HIV diagnosis, current physical and mental health, and psychosocial support are important factors for improving the sexual response. A medical team that is up to date on human sexuality is necessary for the diagnosis and treatment of sexual dysfunctions. When these conditions are preserved, the therapeutic results are good [41–43].

The problem is that it is often the case that sexual issues are not fully investigated by health professionals and few patients will talk about sexual problems spontaneously. As sexual dysfunctions are so prevalent in the general population and in people living with HIV, a lot of them, have not received medical care on their sexual difficulties.

Another important point in the successful diagnosis and treatment of sexual dysfunctions lies in the scarcity of health professionals with expertise in human sexuality. A private issue such as this one needs professionals with the ability to address these sensitive issues with their patients. Otherwise, patients are not likely to disclose their sexual problems to their doctors. When a patient receives medical care on them sexual difficulties, they feels valued and will be more open to engage in adherence to both medications and safer sex strategies.

## 5. Conclusions

Sexual dysfunctions of HIV-positive men are well documented. Several researchers have investigated factors associated with sexual dysfunction in this population. The association between sexual dysfunction and antiretrovirals, particularly protease inhibitors, has been reported in many studies. Some important findings have contributed to understanding the pathophysiological issues associated with sexual dysfunction, but it is still unclear how antiretrovirals cause sexual dysfunctions. The clinical trials show that testosterone replacement is beneficial in terms of sexual functioning. With regard to psychological interventions, there are not enough studies to provide any evidence of a therapeutic effect.

## Figures and Tables

**Table 1:** Associated factors studies.

Study	Population	Method	Results
Mao et al. 2009 [16]	542 gay men, 40% of whom were HIV+	Cross-sectional. Nonstandardized instrument was used	In HIV+ men SD was associated with avoidant strategies to cope with daily life stress, sexual risk-taking in casual encounters, and use of antidepressants

Trotta et al. 2008 [12]	612 HIV+ outpatients using HAART of whom 72% were men	Intercohort analysis 2 Italian cohort studies (AdICoNA; AdeSpall). Global Health Status (GHS) was used	SD associated with suboptimal HAART adherence and self-reported worsening of viro-immunological parameters

Bouhnik et al. 2008 [17]	1,812 HIV+ outpatients (40.6% homosexual men, 24.4% women)	National cross-sectional survey. Nonstandardized instruments were used	Sexual difficulties were associated with discrimination, suffering from lipodystrophy, very disturbing HIV-related symptoms, but were not with HAART

Crum-Cianflone et al. 2007 [18]	300 HIV+ male outpatients	Cross-sectional study. International IIEF, Androgen Deficiency in Aging Men (ADAM Questionnaire) Beck Depression Inventory	ED was associated with older age and depression, not HAART and hypogonadism. Current higher CD4 account was protective against ED.

Asboe et al. 2007 [19]	668 HIV+ male outpatients	Prevalence and associated factors study. Clinical data collected by interview and case review. International Index of Erectile Function (IIEF) was used.	ED: older age, heterosexual, nonalcohol use, depression, antidepressants, psychotropic, and duration of HAART. LSD: older age, depression, and black African ethnicity

Guaraldi et al. 2007 [20]	336 HIV+ male outpatients	Cross-sectional study. Prevalence and associated factors were investigated. IIEF was used. Testosterone level was checked	ED was independently associated with body mass index. Desire, orgasm and satisfaction were associated with mental health scores. Testosterone, metabolic alterations, and HAART were not associated with SD

Richardson et al. 2006 [21]	190 HIV+ male outpatients	Retrospective notes on risk factors to SD written last 18 months. Nonstandardized instruments were used	Retarded ejaculation was associated with peripheral neuropathy

Cove and Petrak, 2004 [3]	78 HIV+ male outpatients	Cross-sectional study. Prevalence and associated factors were investigated. Hospital Anxiety and Depression Scale (HADS).	Risk cognitions such as wanting to lose oneself in sex and leaving responsibility for condom use to the active partner were associated with ED related to condom use, and low T-cell count (<200) was associated with sexual problems

Lamba et al. 2004 [22]	73 HIV+ male (88% MSM) and 100 HIV- MSM	Prospective study. Non-standardized instrument was used	Increased estradiol serum levels and LSD were associated with HAART

Collazos et al. 2002 [23]	189 HIV+ clinically stable outpatient males using antiretrovirals	Prospective study. Nonstandardized instruments were used	Only antiretrovirals remained associated to SD after logistic regression. Hypogonadism was not associated

Collazos et al. 2002b [24]	189 HIV+ clinically stable outpatient males using antiretrovirals	Prospective study. Nonstandardized instruments were used	HAART was associated with increased levels of both testosterone (more with PI) and 17beta-estradiol (more with nonnucleoside reverse transcriptase inhibitors)

Schrooten et al. 2001 [25]	720 male and 184 female HIV+ outpatients	Multicentric cross-sectional study. Anonymous questionnaire. Nonstandardized instruments were used	LSD was associated with protease inhibitors current and past, symptomatic HIV infection, age, and homosexual HIV transmission mode. ED was associated with protease inhibitors, symptomatic HIV, age and tranquilizers

Sollima et al. 2001 [26]	334 HIV+ male outpatients	Cross-sectional study. Beck depression inventory	ED was associated with homosexuality, CD4 cell count, viral load, and indinavir. Protease inhibitors were associated with peripheral neuropathy causing ED

Catalan and Meadows, 2000 [27]	34 gay and bisexual men HIV+ outpatients	Cross-sectional study. Nonstandardized instrument was used	Psychogenic ED associated with nonuse of antiretrovirals

Newshan et al. 1998 [6]	50 HIV+ male outpatients	Cross-sectional study. Derogatis Sexual Functioning Inventory was used	Sexual dissatisfaction was associated with symptomatic HIV/AIDS

Note: SD = sexual dysfunctions.

**Table 2:** Clinical management studies.

Study	Population	Method	Results
Wasserman et al., 2008 [66]	3 HIV+ male with ED, decreased morning erections, and libido, wasting syndrome, and hypogonadism	Case report. All presented low testosterone and SHBG serum levels while using oxandrolone and testosterone	After discontinuing oxandrolone one showed improved libido and ED and all showed improved testosterone and SHBG

Richardson et al., 2007 [70]	13 MSM HIV+ on HAART with LSD and estradiol levels >120 pmol/L	Subjects were randomly allocated to receive testosterone (*N* = 6) or letrozole (*N* = 7) for 6 weeks. Spector Sexual Desire Inventory (SSDI), Depression/Anxiety Stress Scale (DASS) were used	Desire and mean sexual acts improved in both treatment arms. Luteinizing hormone and testosterone increased in all men on letrozole

Schrader et al., 2005 [42]	48 HIV+ males with decreased testosterone levels, libido, and sexual functioning	Multicenter randomized controlled trial. Testim gel was used by 24 men after 2 weeks using AndroGel. Brief male sexual function inventory (BMSFI) was used	Experimental group improved in 4 of 5 domains (sexual drive, erectile function, problem assessment, and sexual satisfaction) of BMSFI (*P* < 0.05)

Catalan and Meadows, 2000 [27]	34 gay and bisexual men HIV+	Cross-sectional study. Nonstandardized instrument was used	Cognitive behavioral therapy, alprostadil, and sildenafil.76% resolved the problem and 14% improved, particularly with physical therapy

Rabkin et al., 2000 [69]	74 HIV+ male outpatients	Double-blind, placebo-controlled 6-week trial with biweekly testosterone injections, followed by 12 weeks. Clinical Global Impressions Scale (CGIS) was used	Testosterone group improved more on libido and energy than placebo group (*P* < 0.05). Parameters were kept by 18 weeks

Wagner and Rabkin, 1998 [45]	23 AIDS eugonadal men with hypogonadism symptoms	Intervention study of 12 weeks of biweekly intramuscular injections of testosterone cypionate CGIS was used	Majority of the 19 subjects who completed the trial showed improvement of SD and other hypogonadism symptoms

Note: AndroGel is a registered trademark of Solvay Pharmaceuticals, Inc., Marietta, Georgia, USA, and Testim is a registered trademark of Auxilium Pharmaceuticals, Inc., Norristown, Pennsylvania, USA. MSM: Men who have sex with men. SD: sexual dysfunctions.

**Table 3:** Clinical differences between organic and psychogenic sexual dysfunction.

Characteristics	Organic	Psychogenic
Age of onset	Older	Younger
Onset	Insidious	Quick
Pattern	Constant	Variable
Masturbation	Yes	No
Adverse life events and/or problems on the onset of sex life	No	Yes
Men: penile nocturnal Erection	No	Yes
